# *AGER* -429T/C Is Associated with an Increased Lung Disease Severity in Cystic Fibrosis

**DOI:** 10.1371/journal.pone.0041913

**Published:** 2012-07-30

**Authors:** Julie Beucher, Pierre-Yves Boëlle, Pierre-François Busson, Céline Muselet-Charlier, Annick Clement, Harriet Corvol, French C F Modifier Gene Study Investigators, M. Abely, C. Belleguic, G. Bellon, K. Bessaci, A.S. Bonnel, F. Brémont, J. Brouard, S. Bui, R. Chiron, R. Chumbi-Flores, A. Clement, H. Corvol, J.C. Dalphin, M.L. Dalphin, V. David, S. de Miranda, J. Derelle, P. Domblides, S. Dominique, J.C. Dubus, I. Durieu, S. Dury, M. Ellaffi, R. Epaud, A. Fanton, M. Fayon, E. Fleurence, P. Foucaud, J.L. Ginies, B. Godbert, D. Grenet, M. Guillot, M. C. Héraud, B. Housset, D. Hubert, F. Huet, R. Kessler, A. Labbé, M. Laurans, M. le Bourgeois, P. le Roux, C. Llerena, G.A. Loeuille, C. Marguet, L. Mely, V. Moisan-Petit, A. Munck, M. Murris-Espin, R. Nove Josserand, J.C. Pautard, I. Pin, S. Pramil, A. Prevotat, G. Rault, P. Reix, N. Remus, M. Renouil, M. Reynaud-Gaubert, B. Richaud Thiriez, M. Roussey, I. Sermet-Gaudelus, N. Stremler, M.L. Uffredi, T. Urban, P. Vigneron, B. Wallaert, L. Weiss

**Affiliations:** American Memorial Hospital, Reims/BASSINET,L., Centre Hospitalier Intercommunal de Créteil, Créteil; Hôpital Pontchaillou, Rennes; Hôpital Femme Mère Enfant, Bron; American Memorial Hospital, Reims; Hôpital André Mignot, Le Chesnay; Hôpital des Enfants de Toulouse, Toulouse; Centre Hospitalier Universitaire de Caen, Caen; Hôpital Des Enfants Groupe Pellegrin, Bordeaux; Hôpital Arnaud de Villeneuve, Montpellier; Hôpital de la Tronche, Grenoble; Hôpital Armand Trousseau, Paris; Hôpital Armand Trousseau, Paris; CNRS-UFC,UMR 6249 Chrono-environnement, Hôpital Jean Minjoz, Besançon; Centre Hospitalier Universitaire de Besançon, Besançon; Hôpital Mère-Enfant, Nantes; Hôpital Foch, Suresnes; Hôpital d’Enfants, Vandoeuvre les Nancy; Hôpital Haut Lévêque, Pessac; Centre Hospitalier Universitaire Charles Nicolle, Rouen; Hôpital d’Enfants de la Timone, Marseille; UCBL1,Groupe Hospitalier Lyon Sud - Hospices Civils de Lyon, Pierre Bénite; Hôpital Maison Blanche, Reims; Centre Hospitalier Universitaire de Caen, Caen; Centre Hospitalier Intercommunal de Créteil, Créteil; Hôpital d’Enfants du Bocage, Dijon; Hôpital Des Enfants Groupe Pellegrin, Bordeaux; Hôpital d’Enfants, Saint-Denis de la Réunion; Hôpital André Mignot, Le Chesnay; Centre Hospitalier Universitaire d’Angers, Angers; Hôpital de Brabois, Vandoeuvre les Nancy; Hôpital Foch, Suresnes; Centre Hospitalier Robert Bisson, Lisieux; Centre Hospitalier Estaing, Clermont-Ferrand; Centre Hospitalier Intercommunal de Créteil, Créteil; Hôpital Cochin, Paris; Hôpital d’Enfants du Bocage, Dijon; Hôpital Civil, Strasbourg; Centre Hospitalier Estaing, Clermont-Ferrand; Centre Hospitalier Universitaire de Caen, Caen; Necker Hôpital d’Enfants Malades, Paris; Hôpital Jacques Monod, Montivilliers; Hôpital de la Tronche, Grenoble; Centre Hospitalier de Dunkerque, Dunkerque; Centre Hospitalier Universitaire Charles Nicolle, Rouen; Hôpital Renée Sabran, Giens; Centre Hospitalier Bretagne Atlantique, Vannes; Hôpital Robert Debré, Paris; Hôpital Larrey, Toulouse; Groupe Hospitalier Lyon Sud - Hospices Civils de Lyon, Pierre Bénite; Hôpital Nord, Amiens; INSERM U823 Université Joseph Fourier,Hôpital de la Tronche, Grenoble; Centre Hospitalier Universitaire Charles Nicolle, Rouen; Hôpital Calmette, Lille/RAMES,C., Hôpital Nord, Amiens; Centre de Perharidy, Roscoff; Hôpital Femme Mère Enfant, Bron; Centre Hospitalier Intercommunal de Créteil, Créteil; Groupe Hospitalier Sud Réunion, Saint-Pierre de la Réunion; Hôpital Nord, Marseille; Hôpital Jean Minjoz, Besançon; Université de Rennes 1,Hôpital Sud Annexe Pédiatrique,Rennes; Necker Hôpital d’Enfants Malades,Paris; Hôpital d’Enfants de la Timone, Marseille; Centre Hospitalier Bretagne Atlantique, Vannes; Centre Hospitalier Universitaire d’Angers, Angers; Centre Hospitalier Bretagne Sud, Lorient; Hôpital Calmette, Lille; Hôpital de Hautepierre, Strasbourg; 1 AP-HP, Hôpital Trousseau, Pediatric Pulmonary Department, Inserm U938, Paris, France; 2 CHU de Rennes, Hôpital Sud, Rennes, France; 3 Université Pierre et Marie Curie-Paris 6, Paris, France; 4 AP-HP, Hôpital St Antoine, Biostatistics Department, Inserm UMR-S707, Paris, France; The Chinese University of Hong Kong, Hong Kong

## Abstract

The clinical course of cystic fibrosis (CF) varies between patients bearing identical *CFTR* mutations, suggesting the involvement of modifier genes. We assessed the association of lung disease severity with the variant *AGER* -429 T/C, coding for RAGE, a pro-inflammatory protein, in CF patients from the French CF Gene Modifier Study. We analyzed the lung function of 967 CF patients *p.Phe508del* homozygous. FEV_1_ was analyzed as CF-specific percentile adjusted on age, height and mortality. *AGER* -429T/C polymorphism was genotyped and its function was evaluated *in vitro* by measurement of the luciferase activity. *AGER* -429 minor allele (C) was associated with poorer lung function (p = 0.03). *In vitro*, the promoter activity was higher in cells transfected with *AGER* -429C compared to cells transfected with the *AGER* -429T allele (p = 0.016 in BEAS-2B cells). *AGER* seems to be a modifier gene of lung disease severity in CF, and could be an interesting biomarker of CF airway inflammation. The functional promoter *AGER* -429C variant is associated with an increased RAGE expression that can lead to an increased lung inflammation and a more severe lung disease.

## Introduction

Cystic fibrosis (CF) is the most common severe autosomal recessive genetic disease in Caucasians caused by mutations in the *CFTR* (cystic fibrosis transmembrane conductance regulator) gene. In patients with CF, lung disease is the major cause of morbidity and mortality. The progressive decline of pulmonary function is due to a vicious cycle of airways infection and inflammation. The inflammatory process in CF lung is dominated by a neutrophilic influx, associated with high concentrations of neutrophil-derived mediators, in particular pro-inflammatory cytokines such as the interleukin (IL)-8 and the receptor for advanced glycation endproducts (RAGE) [1], [2].

RAGE is a member of the cell surface receptor immunoglobulin superfamily. It is a multiligand receptor with a short cytosolic domain and a large extra cellular region containing 3 immunoglobulin-like-domains (V, C1, C2 domains). These domains co-ordinately interact and bind with different ligands, leading to the activation of several proinflammatory signalling pathways [3]. RAGE is expressed in almost all tissues, although the highest levels are found in the respiratory alveolar type I epithelial cells [4], [5]. It is well described that RAGE plays a major role in lung homeostasis [4], [6]. In pulmonary diseases, RAGE has been shown to be either increased or decreased [2], [7], [8]. Increased levels of RAGE have been observed in the alveolar walls of patients with chronic obstructive pulmonary disease (COPD) compared to controls [8]. In CF, RAGE expression has been shown to be up-regulated in the CF airway neutrophils compared to their blood counterparts [2]. On the opposite, loss of RAGE has been incriminated in idiopathic pulmonary fibrosis pathogenesis [7].

The gene encoding RAGE, *AGER*, is highly polymorphic. Several variants have been described with various functional effects [9], [10]. Interestingly, recent genome-wide association studies (GWAS) conducted in huge European cohorts have shown that one *AGER* variant (rs2070600) was correlated with lung function [11], [12]. Moreover, we and other have previously shown that the common ancestral haplotype 8.1, which contains the promoter variant *AGER* -429T/C (rs1800625), was associated with lung disease severity in European CF patients [13], [14].

Based upon the biologic, genetic and clinical evidence, we hypothesized that the variant *AGER* -429 T/C was involved in the exuberant lung inflammation process in CF, which drives the decline of lung function. To investigate this hypothesis, we first tested whether this variant was associated with lung disease severity in CF patients from the French CF Gene Modifier Study. In addition, we tested whether this promoter variant was associated with a modulation in RAGE expression.

## Results

### Clinical Characteristics of the CF Patients from the French CF Gene Modifier Study and *AGER* -429T/C Distribution

The main characteristics of the study population are listed in Table 1. As required by the inclusion criteria, the 967 patients were from European origin, *CFTR* p.Phe508del homozygous and pancreatic insufficient. Median age was 19.8 years. Minor allele frequency of *AGER*-429 T/C variant was similar to that observed in Caucasians (14.5% *vs*. 15.5% in Hapmap) and did not show evidence of HWE departure (p = 0.52).

**Table 1:** Clinical characteristics of the CF subjects from the French CF Gene Modifier Study overall and according to AGER -429T/C distribution.

	All	AGER -429CC carriers	AGER -429CT carriers	AGER -429TT carriers
Study population, n	967	23	235	709
Current age: median [range]	19.8 [6.1–55.6]	18.5 [7.0–55.6]	19.8 [6.1–46.4]	19.8 [6.1–55.1]
Gender (% female)	48%	52%	48%	47%
BMI z-score, median [range]	−0.76 [−6.14–2.08]	−0.61 [−2.05–0.66]	−0.74 [−3.51–1.86]	−0.77 [−6.14–2.08]
FEV1% predicted, median [range]	62.7 [2.8–208]	60.7 [12.9–143.1]	60.2 [12.0–180.8]	64.5 [2.8–208]
Kulich, Z-score predicted, median [range]	−0.07 [−2.33–2.33]	−0.08 [−2.33–2.33]	−0.24 [−2.33–2.33]	−0.01 [−2.33–2.33]
KNoRMA, Z-score predicted, median [range]	0.3 [−1.79–3.21]	−0.03 [−1.56–3.21]	0.16 [−1.71–2.65]	0.34 [−1.79–2.83]

BMI: body mass index, FEV_1_: forced expired volume in 1 second.

### *AGER* -429T/C Association with Lung Disease Severity

**Figure 1:**
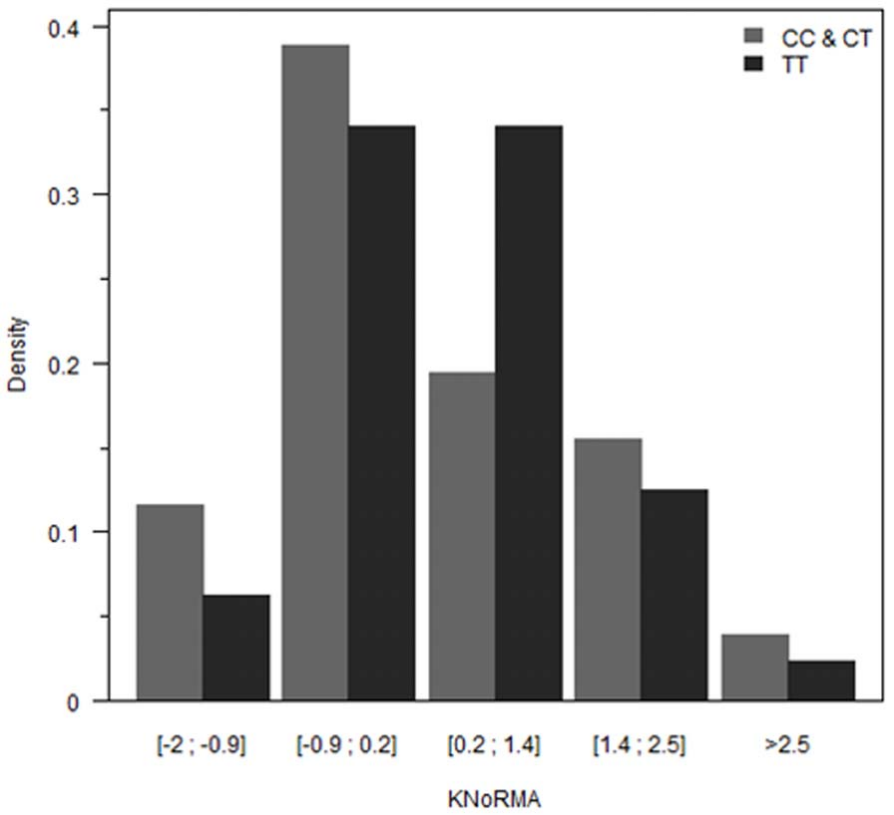
FEV_1_ survival adjusted CF specific percentiles (KNoRMA) distribution in CF subjects according to *AGER* -**429 T/C genotype.** Distribution of the lung function according to the FEV_1_ adjusted on the age, the height and the mortality; expressed in KNoRMA in *AGER* -429 CC and CT carriers (light-grey bars, n = 258) and *AGER* -429 TT carriers (dark-grey bars, n = 709).

The KNoRMA distribution according to the *AGER* -429 T/C genotype is shown in Figure 1 [15]. Compared to the patients homozygous for the major allele (*AGER* -429TT), the patients carrying at least one minor allele (*AGER* -429CC and *AGER* -429 CT*)* had a more severe lung disease severity, manifesting as a significantly lower FEV_1._ This association was observed for each FEV_1_ analyzes: decrease of the mean FEV_1_ percent-predicted (mean difference –4.4±1.9%, p = 0.02), lower Kulich CF-specific percentile Z-score (mean difference: −0.17±0.08, p = 0.03), and smaller KNoRMA (mean KNoRMA difference: −0.15±0.07, p = 0.03).

### Effects of *AGER*-429 T/C Gene Polymorphism on AGER Promoter Activity

**Figure 2:**
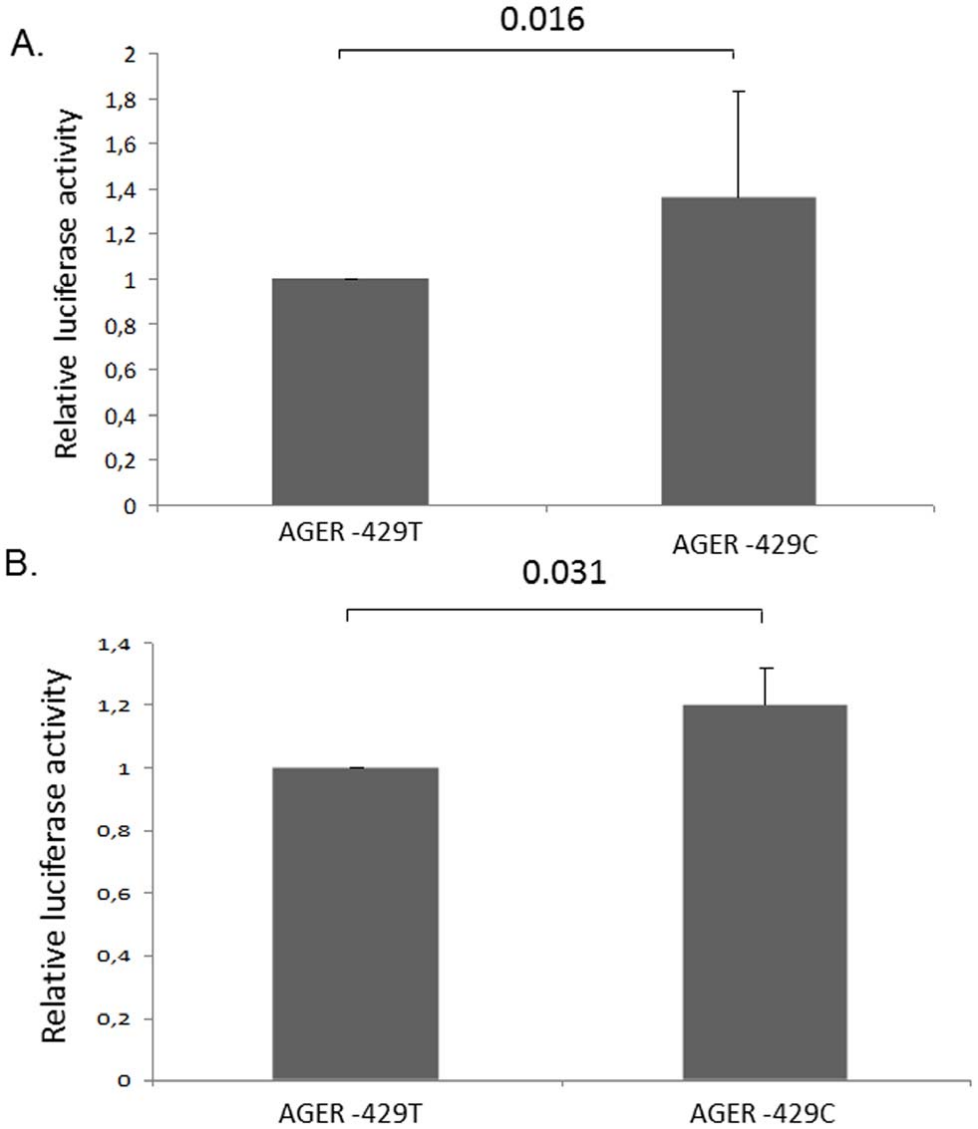
*In vitro* influence of *AGER* -**429T/C polymorphism on the promoter activity.** Constructs containing either *AGER*-*429C* or *AGER*-*429T* and a luciferase reporter gene were transfected into BEAS-2B (A) and A549 (B) cells. Luciferase activity assays were performed in triplicate in six independent experiments. Relative luciferase activity is represented as a ratio against the luciferase activity in cells transfected with the AGER-429T plasmid. Luciferase activity, reflecting activity of the *AGER* gene promoter, was significantly higher in cells containing *AGER*-*429C* plasmid compared to the cells containing *AGER*-*429T* plasmid (p = 0.016 and 0.031 respectively in BEAS-2B (A) and A549 (B) cells).

To determine if *AGER*-*429T/C* modulated the activity of *AGER* promoter, constructs containing either *AGER*-*429C* or *AGER*-*429T* and a luciferase reporter gene were transfected into BEAS-2B and A549 cells. Luciferase activity, reflecting *AGER* gene promoter activity, was significantly higher in cells containing *AGER*-*429C* plasmid compared to the cells containing *AGER-429T* plasmid in both cell lines (Figure 2; p = 0.016 in BEAS-2-B cell line and p = 0.031 in A549 cell line).

## Discussion

This study demonstrated that *AGER* could be a modifier gene of lung disease severity in CF. We observed that the promoter *AGER* -429T/C was associated with CF lung disease severity in a large homogeneous cohort of CF patients and was further able to modulate RAGE expression *in vitro.*

We observed an increase in lung disease severity in CF patients carrying at least one *AGER* -429C allele. The patients who contributed to this study constituted a large and homogeneous cohort, thanks to the participation of most of the French CF centers. This study was indeed an ancillary project of a national program on CF modifier genes. So far, more than 3,500 CF patients have been included in this program, that represented around 50% of the French national CF coverage [16]. Only patients from European origin and homozygous for the most frequent *CFTR* p.Phe508del mutation were selected for this study to limit potential biases driven by ancestry and *CFTR* mutations. Moreover, only patients with lung function measurements (older than 6 years) were kept to allow lung disease severity analyses. Nevertheless, almost 1000 CF patients contributed to the study, constituting a large and homogeneous cohort of patients with this rare disease.

One other originality of this study was the several analyzes applied to appreciate lung disease severity in the CF patients. In CF, lung function, and specifically FEV_1_, decrease remains the main indicator of lung disease severity. FEV_1_ is generally expressed as a percentage of a predicted value based on sex, age, and height in individuals from a healthy reference population [17]. It has been recently recognized that these values were not informative enough for CF [15], [18]. It is indeed obvious that a CF patient with 30% FEV_1_ of the reference at age 8 is much more severe than a patient with 30% FEV_1_ of the reference at age 30. North-American CF specific reference equations have been proposed, by which the FEV_1_ of an individual with CF is compared not to that of a healthy reference population but rather to that of a “typical” CF population [18]. A further modification has been applied to obtain quantitative trait measurements corrected for attrition due to patient mortality with age [15], and usable in genetic association studies [19], namely the KNoRMA. The use of this recently proposed trait quantification procedure allows direct comparison of CF patients, irrespective of age and taking into account mortality. It is therefore an improvement over the previous approaches for comparing lung function.

We previously reported that an ancestral haplotype (8.1 AH), very common in Europeans, was associated with lung function severity in European CF patients [13]. This ancestral haplotype is tagged by several SNPs found near or within genes involved in the inflammatory response, including the presently studied *AGER* -429 T/C polymorphism. However, this difference in severity could have been biased due to pooling CF patients with different *CFTR* genotypes. Our present result, obtained using patients homozygous for the *p.Phe508del CFTR* mutation only and all of European origin, limits the effect of such biases and highlights the role of *AGER* gene. Importantly, we found that more severe lung disease was found in those carrying the -429C polymorphism, the variant that tags the ancestral haplotype. Variants in *AGER* gene have been described to modify the lung function in several genome-wide association studies (GWAS) in large European general population cohorts [11], [12]. This would suggest that genes found to modulate lung function in general population studies should be systematically considered and further tested for their effect in CF, or could even be used to prioritize the analysis of GWAS in CF studies.

We further observed that the *AGER* -429T/C variant, located in the gene promoter, was involved in the regulation of RAGE expression. This observation derived from *in vitro* functional assays using bronchial and alveolar epithelial cells. We have shown that *AGER-429C* was associated with an increased promoter activity, which may lead to an increased RAGE expression. This result is concordant with observations of Hudson *et al.* who suggested that the -429C allele upregulated RAGE expression [20]. RAGE triggers the generation of reactive oxygen species and the activation of signal transduction pathways such as NF-κB, AP-1 and mitogen activated protein kinases (MAPKs), all known to be dysregulated in CF epithelial cells [21], [22]. Consequently, we could hypothesize that the excess of RAGE, observed in presence of the -429C allele, could lead to an increased airway inflammation, enhancing the lung disease severity. Further studies would be required to confirm the observed results in CF cell lines.

To conclude, these findings suggest that RAGE could be an interesting biomarker of lung disease severity in CF. The functional promoter -429C variant in the gene encoding RAGE is indeed associated with an increased RAGE expression. Further studies are needed to confirm that this variant can lead to an increased lung inflammation in CF and, consequently, to a more severe lung disease.

## Materials and Methods

### Ethic Statement

The study was approved by the French ethical committee (Comité de Protection des Personnes CPP n°2004/15) and the information collection was approved by the CNIL (Commission Nationale de l’Informatique et des Libertés n°04.404).

### Study Populations

The 49 French CF centers care for an estimated 5,000 to 6,000 CF patients. In 2006, prospective enrollment of prevalent and incident CF patients was initiated by the French CF Gene Modifier Consortium from 38 out of the 49 French CF centers. We selected for *AGER* -429 T/C genotyping 967 CF patients who were over 6 years of age, had both parents born in a European country and were homozygous for the *CFTR* p.Phe508del mutation. Patients, parents or guardians signed an informed consent form for participation in the study as required by French regulations. Phenotypic informations were obtained from hospital records and collected by a single physician, blinded to the results of patients’ genotype. This included sex, age, *CFTR* genotype and forced expiratory volume in one seconde (FEV_1_) measurements over the last 5 years.

### Quantitative Phenotype Score for Lung Function in CF Patients

A common problem in the comparison of lung disease CF severity between patients is to properly quantify the phenotype. Indeed, FEV_1_ percent predicted, a commonly used approach, may mask differences as it is calculated by reference to a normal population [17]. Furthermore, age adjustment remains necessary as no correction is made for patient selection with age due to mortality. Taylor *et al.* recently introduced the KNoRMA, or “Kulich normal residual mortality adjusted”, as a quantitative phenotype for use in quantifying CF lung function severity [15]. In short, this approach is based on first determining the age and height adjusted CF specific FEV_1_ percentile as in Kulich *et al.* [18], so that each patient is ranked among peers of the same age by a score K between 0 and 100%; and then applying a further correction for attrition due to mortality as S(a)* K + (1-S(a)) where S(a) is the % survival of CF patients at age a. KNoRMA is the inverse normal transformation of this quantity, interpreted as a z-value [15].

### Genotyping

Genotyping of *AGER* -429T/C (rs1800625) was performed by real time polymerase chain reaction (PCR), using the Step-One-Plus real time PCR (Applied Biosystems, USA) and the conventional Taqman primers and probe (Applied Biosystems, Foster City, USA). Allelic discrimination was realized by endpoint measurements with specific fluorescent oligonucleotides (detection system software of the ABI prism 7000).

### Promoter Functional Assay

The functional role of -429T/C in the *AGER* gene was tested by the dual luciferase assay. A 500-bp fragment of the *AGER* promoter was prepared in a luciferase reporter construct (switchgear genomics-S722831). Construct with substitution at nucleotide -*429* was generated by using the QuikChange site directed mutagenesis kit (Stratagene). Construct was generated by amplifying the *AGER* gene by PCR (forward primer 5′-aaatgattttctttcacgaag**c**tccaaacaggtttctctcctg-3′ and reverse primer 5′-caggagagaaacctgtttgga**g**cttcgtgaaagaaaatcattt-3′). The constructs were then amplified in One Shot® TOP10 Chemically Competent *E. coli* (invitrogen) to obtain high-copy number plasmids. The mutagenesis procedure was controlled by DNA sequencing of the constructs.

Human bronchial epithelial cells, BEAS-2B (ATCC, CRL-9609), and adenocarcinomic alveolar basal epithelial cells, A549 (ATCC, CCL-185) cultivated in F-12 Nutrient Mixture with L-glutamine (Invitrogen) containing 10% fetal calf serum and 100 U/mL penicillin G-streptomycin were incubated at 37°C in 5% CO_2_ in a humidified incubator. We seeded 10^5^ cells in each well of 12 plates for 1 day before transfection to obtain 70–80% cells’ confluence. The cells were transfected with the *AGER* plasmid (1 µg) and internal Renilla luciferase control plasmid (100ng) using lipofectamine as transfection reagent (Invitrogen).

Eighteen hours after transfection, cell lysates were prepared with passive lysis buffer (Promega), from the 100-µl lysate, a 20-µl aliquot was assayed for firefly luciferase and Renilla luciferase activity using a Dual-Luciferase Reporter Assay Kit (Promega). The activity of each promoter was directly measured by the ratio of the firefly luciferase level to the Renilla luciferase level. In BEAS-2B and A549 cells, six independent transfection experiments were performed in triplicate.

### Statistical Analysis

Conformance of the allele frequencies with the Hardy-Weinberg equilibrium (HWE) was tested using Fisher’s exact test.

We used linear mixed models of FEV_1_ measurements according to age to obtain patient specific smoothed FEV_1_ prediction at the current age, thereby fully using the longitudinal data to reduce measurement error. This smoothed FEV_1_ value was transformed into percent predicted as described by Knudson *et al.* [23]; into CF-specific percentiles as described by Kulich *et al.* [18] and into KNoRMA value as described by Taylor *et al.* [15].

The association between *AGER* -429 T/C genotype and lung function was then assessed using a linear regression model, with FEV_1_ transformed as described above as the dependent variable. As the frequency of AGER -429 C homozygous carriers was law (n = 23, 8.9%), we adopted a dominant coding for genotype information, testing the effect of the carriage of at least one minor allele and comparing CC/TC versus TT carriers.

Allele-specific differences in luciferase activity were compared using the Wilcoxon signed-rank test. All analyses were done using the R software. A P-value of less than 5% was interpreted as evidence of a statistically significant difference or association.
